# An Omics Approach to Extracellular Vesicles from HIV-1 Infected Cells

**DOI:** 10.3390/cells8080787

**Published:** 2019-07-29

**Authors:** Robert A. Barclay, Pooja Khatkar, Gifty Mensah, Catherine DeMarino, Jeffery S. C. Chu, Benjamin Lepene, Weidong Zhou, Patrick Gillevet, Bahareh Torkzaban, Kamel Khalili, Lance Liotta, Fatah Kashanchi

**Affiliations:** 1 Laboratory of Molecular Virology, George Mason University, Manassas, VA 20110, USA; 2 Applied Biological Materials Inc., 1-3671 Viking Way, Richmond, BC V6V 2J5, Canada; 3 Ceres Nanosciences Inc., Manassas, VA 20110, USA; 4 Center for Applied Proteomics and Molecular Medicine, George Mason University, Manassas, VA 20110, USA; 5 Microbiome Analysis Center, George Mason University, Manassas, VA 20110, USA; 6 Center for Neurovirology, Temple University, Philadelphia, PA 19122, USA

**Keywords:** HIV-1, extracellular vesicle, proteomics, RNA sequencing

## Abstract

Human Immunodeficiency Virus-1 (HIV-1) is the causative agent of Acquired Immunodeficiency Syndrome (AIDS), infecting nearly 37 million people worldwide. Currently, there is no definitive cure, mainly due to HIV-1′s ability to enact latency. Our previous work has shown that exosomes, a small extracellular vesicle, from uninfected cells can activate HIV-1 in latent cells, leading to increased mostly short and some long HIV-1 RNA transcripts. This is consistent with the notion that none of the FDA-approved antiretroviral drugs used today in the clinic are transcription inhibitors. Furthermore, these HIV-1 transcripts can be packaged into exosomes and released from the infected cell. Here, we examined the differences in protein and nucleic acid content between exosomes from uninfected and HIV-1-infected cells. We found increased cyclin-dependent kinases, among other kinases, in exosomes from infected T-cells while other kinases were present in exosomes from infected monocytes. Additionally, we found a series of short antisense HIV-1 RNA from the 3′ LTR that appears heavily mutated in exosomes from HIV-1-infected cells along with the presence of cellular noncoding RNAs and cellular miRNAs. Both physical and functional validations were performed on some of the key findings. Collectively, our data indicate distinct differences in protein and RNA content between exosomes from uninfected and HIV-1-infected cells, which can lead to different functional outcomes in recipient cells.

## 1. Introduction

Human Immunodeficiency Virus-1 (HIV-1) is the causative agent of Acquired Immunodeficiency Syndrome (AIDS), a significant concern infecting 37 million worldwide and over 1 million in the United States [1,2,3]. With the aid of combination antiretroviral therapy (cART), it is possible to inhibit viral replication and control infection, greatly increasing life expectancy and overall quality of life. Antiretroviral drugs prevent viral spread through a number of mechanisms, including by blocking new infection and by inhibiting viral maturation in infected cells [4]. This promotes latency, or a state in which viral particles are below the detection limit (<50 copies/mL) [5]. However, cART does not eliminate or cure the viral infection as it is unable to eradicate latent viral reservoirs, evidenced by rebounding viral titers and progression of disease in patients who do not adhere to cART [6,7,8,9,10]. Furthermore, recent literature has challenged the notion that cART allows for a true, transcriptional latency as studies have shown that patients under cART still have a large buildup of HIV-1 RNA within infected cells and production of defective and/or mutant viruses over time, potentially contributing to complications such as HIV-1 Associated Neurocognitive Disorder (HAND) [11,12,13,14,15]. Interestingly, these viral noncoding RNAs may be exported from infected cells through extracellular vesicles (EVs), including exosomes [16,17,18].

Exosomes are a type of extracellular vesicle of about 30–120 nm in diameter found throughout various body fluids, such as urine and cerebral spinal fluid (CSF) [19,20,21,22]. These vesicles form within multivesicular bodies in the late endosome and are typically associated with the Endosomal Complex Required for Transport (ESCRT) pathway [23]. Consequently, exosomes can be enriched with certain proteins associated with ESCRT, including TSG-101 and Alix, along with members of the tetraspanin family, such as CD63 [24].

Surrounded by a lipid bilayer of approximately 5 nm in thickness, exosomes were originally characterized in 1991 and thought to be a means for cells to discard waste [25]. However, exosomes are now known to carry cargo between cells, including proteins and RNA [20,26]. This can elicit a cellular response in the target cell (recipient cell), which includes transcription, translation, and immune modulation; depending upon the condition of the originating cell (donor cell) at the time, the effects in the recipient cell can either be beneficial or deleterious [16,17,20,22,27,28].

Exosomes have been shown to play a role in HIV-1 pathogenesis. Studies have shown that exosome-associated HIV-1 Nef was able to elicit TNF-α production in recipient peripheral blood mononuclear cells (PBMCs) and induce bystander apoptosis in recipient CD4^+^ T-cells [29,30]. Exosomes from HIV-1-infected cells have also been reported to disrupt the blood/brain barrier (BBB) in an in vitro model [31]. A 2014 study showed that exosomes from HIV-1-infected cells promoted HIV-1 replication in recipient cells [32]. Additionally, we have previously shown that exosomes originating from infected cells contain TAR RNA, which inhibits apoptosis, shuts down the PKR/eIF2α pathway, and activates TLR-3 and the NF-κB pathway in uninfected recipient cells [16,17]. Furthermore, it has been suggested that exosomes from HIV-1-infected dendritic cells are able to mediate viral trans-infection via fibronectin and galectin-3 [33]. Additionally, exosomes from both uninfected and infected cells have been demonstrated to activate HIV-1 from latency in infected cells [27,34]. This led us to ask about the RNA and protein contents included in exosomes that could be associated with HIV-1 and functional effects within recipient cells.

Our current study aims to address two main points regarding exosomes and their content. First, we performed both proteomics and RNA sequencing on exosomes originating from both uninfected and HIV-1-infected T-cells and monocytes. Second, we examined whether DNA was present in the exosomes. We found that exosomes from infected T-cells were enriched in histones and also contained numerous cyclin dependent kinases (Cdks) and Src family kinases that were absent from uninfected cell exosomes. We also observed several long noncoding RNAs in exosomes from infected T-cells that were missing in monocytes. Furthermore, mitochondrial DNA was found in large EVs from both uninfected and infected cells. Our findings suggest that infection with HIV-1 may alter the contents of the cargo packaged into exosomes originating from infected cells, leading to different functional effects within recipient cells.

## 2. Materials and Methods

### 2.1. Cells and Treatment of PBMCs

CEM (uninfected T-cells), ACH2 (HIV-1-infected T-cells), J1.1 (HIV-1-infected T-cells), U937 (uninfected promonocytic cells), U1 (HIV-1-infected monocytes), and OM10.1 (HIV-1-infected myeloid-derived cells) cells were grown in complete RPMI media at 37 °C and 5.0% CO_2_. PBMCs were also cultured in complete RPMI media at 37 °C and 5.0% CO_2_. PHA and IL-2 were added to the PBMCs every other day over the course of 7 days. Then, the supernatant was collected and PBMCs were infected with HIV-1 89.6 (MOI: 10) and again treated with PHA/IL-2. Three days after infection, PBMCs were treated with a cART cocktail of indinavir, lamivudine, emtricitabine, and tenofovir (each component was at 10 µM) and IL-7, followed by treatment with cART and IL-7 every other day for 6 days to promote latency. The role of IL-7 in combination with cART is to move T-cells to the quiescent phase in order to decrease the level of HIV-1 virion production [17]. Day 6 post infection, the supernatant was collected. Bovine exosomes in the fetal bovine serum (FBS) were excluded from all culture media by ultracentrifugation at 100,000× *g* for 90 min prior to addition to RPMI media. All cells infection with HIV-1 89.6 were performed in a limited-access, separate BSL-2.5 space designated for HIV-1 work. Ultracentrifugation of supernatant from HIV-1 infected cells was performed in a separate space away from the main laboratory environment as well.

### 2.2. Reagents and Antibodies

Complete culture media consisted of RPMI 1640 or D-MEM supplemented with 10% FBS, 1% L-glutamine, and 1% streptomycin/penicillin. Antibodies used for Western blot assays were HSP70 (sc-1060-R), Cdk2 (78B2), Cdk9 (C12F7), PKR (sc-707), hnRNPA2/B1 (sc-53531), histone H1 (sc-8030), and actin (ab-49900). Antibodies against Cdk2 and Cdk9 utilized 1:1000 dilutions while 1:250 dilutions were utilized for antibodies against HSP70, PKR, hnRNPA2/B1, and histone H1. A 1:5000 dilution was utilized for the antibody against actin.

### 2.3. Nanotrap^®^ Particle Pulldown

The Nanotrap^®^ (NT) particles utilized here have been described in detail previously [35]. For the capture and isolation of EVs from cell culture supernatants, a 25 µL slurry (30%) of phosphate-buffered saline without calcium and magnesium (PBS), CN1030 (NT80 red), and CN2010 (NT82 blue) NT particles (Ceres Nanosciences) was incubated with 1 mL of cell supernatant. Samples were rotated at 4 °C for 24–72 h to allow for NT particle capture. NT pellets were isolated and washed with sterile PBS before preparation for the downstream assays.

### 2.4. EV Isolation

Cells were grown in appropriate media, supplemented with 10% FBS. Ten milliliters of cell culture supernatants (produced from a culture of one million cells per mL for 5 days) were pelleted by centrifugation at 300× *g* and 4 °C for 10 min to remove cells. The supernatant was transferred to a clean, sterile tube, and an equal volume of a polyethylene glycol-based reagent, ExoMAX (SBI), was added to the supernatant and allowed to incubate overnight at 4 °C. This was followed by a 30 min spin at 1500× *g* at room temperature to pellet vesicles. The supernatant was discarded and the vesicle pellet was re-suspended in 300 μL of PBS.

### 2.5. EV Purification

Iodixanol (OptiPrep^TM^) gradients were prepared in PBS in 1.2% increments ranging from 6% to 18%. Vesicles isolated using ExoMAX (300 μL) were layered on top of the gradient and ultra-centrifuged for 90 min at 100,000× *g* in a SW41 Ti rotor (Beckman). Gradient fractions were collected from the top of the gradient in 1 mL increments and transferred to sterile 1.5 mL centrifuge tubes. A 30% Nanotrap^®^ particle slurry of CN1030/CN2010 (NT80/82) (Ceres Nanosciences) was added to each fraction, and Nanotrap^®^ particle pulldown was performed as described above. The 10.8 and 12.0 fractions were used for proteomics analysis. The aforementioned fractions used were free from virus, as described previously [36]. All centrifugations were performed at 4 °C.

### 2.6. ZetaView Nanoparticle Tracking Analysis 

NTA was performed using the ZetaView^®^ Z-NTA (Particle Metrix, Inning am Ammersee, Germany) and its corresponding software (ZetaView 8.04.02). One hundred nanometer polystyrene nanostandard particles (Applied Microspheres) were used to calibrate the instrument prior to sample readings at a sensitivity of 65 and a minimum brightness of 20. Automated quality control measurements including, but not limited to, cell quality check and instrument alignment and focus were also performed prior to the use of the ZetaView for sample measurements. For each measurement, the instrument pre-acquisition parameters were set to a temperature of 23 °C, a sensitivity of 85, a frame rate of 30 frames per second (fps), and a shutter speed of 250. For each sample, 1 mL of the sample, diluted in PBS, was loaded into the cell, and the instrument measured each sample at 11 different positions throughout the cell, with three cycles of readings at each position. After automated analysis and removal of any outliers from the 11 positions, the mean, median, and mode (indicated as diameter) sizes, and the concentration of the sample, were calculated by the machine software. Measurement data from the ZetaView were analyzed using the corresponding software, ZetaView 8.04.02, and Microsoft Excel 2016. We selected the mode, defined as the size of the most abundant particles, as the measurement for size in our analysis. 

### 2.7. Mass Spectrometry

EVs from NT particle processed 10.8 iodixanol fraction (see above) was lysed by treatment with 8M urea. Ten millimolar DTT was used to reduce samples, which were then alkylated with 50 mM iodoacetamide. The samples were then diluted by a solution of equal parts water and 500 mM NH_4_HCO_3_. Samples were then digested with trypsin (Promega) for 4 h at 37 °C. Samples were then centrifuged at 12,000× *g* at room temperature for 10 min to remove Nanotrap^®^ particles, and the supernatants were collected into a microcentrifuge tube and frozen overnight at −20 °C. ZipTip was then used to collect the peptide samples, which were dried and re-suspended in 10 μL of 0.1% TFA solution, prior to loading into an Orbitrap Fusion mass spectrometer. Bioinformatic searches from Swiss-Prot were used to identify peptides, and a label-free precursor ion detection method (Proteome Discoverer, version 1.3; Thermo Fisher Scientific, Waltham, MA, USA) was used for accurate mass measurements on proteins/peptides with specific retention times on precursors/fragments. 

### 2.8. [^3^H] Thymidine DNA Synthesis Assay

EVs from the 10.8 fraction of CEM, ACH2, U937, and U1 cells were added to CEM or U937 cells grown in the appropriate media with 0.1% FBS to ensure cells were synchronized in G0 phase. Forty-four hours after incubation with exosomes at 37 °C, cells were treated with [^3^H] thymidine at a concentration of 1 µCi/well and incubated for 4 h at 37 °C. The cells were then washed twice with cold PBS and prefixed for 3 min with a formulation of 1:1 PBS/fixative (70% ethanol/30% acetic acid). Cells were fixed in the fixative solution for 20 min at 37 °C followed by a treatment with 0.3 M perchloric acid for 10 min. Following fixation, the cells were washed with distilled water and then incubated for 20 min with 100 µL of 0.25 M sodium hydroxide. An equal volume of distilled water was added prior to transfer of the hydrolysate to scintillation vials with 3 mL of scintillation fluid. Cells were counted for 5 min on a beta-counter.

### 2.9. Protein-Protein Interactions (PPIs) Analysis

Search Tool for the Retrieval of Interacting Genes (STRING) is a web-open database of protein-protein interactions (PPIs). STRING (version 10.5) as of now covers 9,643,763 proteins from 2,031 living beings, including *Homo sapiens*. To assess the protein relationship among EVs from uninfected (CEM)/infected (ACH2) T-cells and EVs from uninfected (U937)/infected (U1) monocytes, we mapped the mass spectrometry retrieved proteins from EVs of each cell line to STRING; interactions with a combined score ≥ 0.70 (high confidence) were considered significant. In the resulting protein association network, proteins are presented as nodes which are connected by lines, where thickness represents the confidence level (0.7–0.9) and color represents the prediction mechanism.

### 2.10. Western Blot Analysis

Samples were loaded onto a 4–20% Tris-glycine gel (Invitrogen) and run at 180V. An overnight transfer of proteins to Immobilon membranes (Millipore) at 50mA was then performed. Membranes were then blocked for 30 min with PBS containing 0.1% Tween 20 (PBS-T) and 5% dry milk at 4 °C. Membranes were incubated overnight at 4 °C with the appropriate primary antibody against specified proteins. The next day, membranes were washed twice with PBS-T and incubated with appropriate HRP-conjugated secondary antibody in PBS-T for 2 h at 4 °C. Membranes were then washed twice with PBS-T and once with PBS. Membranes were developed with Clarity Western ECL Substrate (Bio-Rad) and visualized by the Molecular Imager ChemiDoc Touch system (Bio-Rad). 

### 2.11. RNA and DNA Isolation

CEM, Jurkat, U937, ACH2, J1.1, U1, HUT-102, and MT-2 cell supernatant was treated with NT80/NT82 as described above. For RNA isolation, the resulting pellet was RNA was isolated directly off the pellet using TRI Reagent-LS (MRC) according to the manufacturer’s protocol. For DNA isolation, the NT pellet was washed and suspended in PBS and treated with DNAse (50 ng) and RNAse A (100 ng) for 1 h at 37 °C to remove extra-EV nucleic acid contaminants. The PBS was aspirated and the NT pellet re-suspended in TNE50 with 0.1% NP40 with RNaseA (100 ng) for one hour at 37 °C to remove EV-associated RNAs. An equal volume of phenol/chloroform with isoamyl alcohol was added and the samples were spun at 14,000 rpm for 10 min. The top layer was transferred to a new tube, an equal volume of chloroform was added, and the samples were again spun at 14,000 rpm for 10 min. Again, the top layer was collected into a new tube (this was to ensure there was no phenol contamination). A 1/10^th^ volume of sodium acetate (3 M) was added to each sample, followed by an equal volume of isopropanol, and DNA was precipitated overnight at −70 °C. The samples were then spun at 14,000 rpm for 15 min, the resulting pellet washed with 70% ethanol, and the DNA dried using a vacuum dryer. The DNA was then re-suspended in 20 μL of nuclease free water. 

### 2.12. RNA Sequencing Library Construction

RNA processing and library construction was adapted from our previous work [27]. Briefly, the 3′-end of the extracted exosomal RNA was processed using a miRNA cDNA synthesis kit (Applied Biological Materials, cat. no. G902). A poly(A) tail was added to the transcripts using Poly(A) polymerase followed by first strand synthesis using an oligo(dT) adapter. Second strand synthesis was performed using TAR-sequence adapter and amplified with 3′-adpater-specific primer. The amplified DNA was purified using DNA purification SPRI Magnetic Beads (Applied Biological Materials, cat. no. G950). The cDNA was fragmented using Fragmentase (NEB) followed by ligation of Illumina sequencing adapters for construction of the sequencing library. Quality control of the sequencing library (which included nucleotide length assessment by RNA Agilent Trace) was performed using Agilent 2100 Bioanalyzer (Agilent) and qPCR according to the manufacturer’s instructions. Sequencing was performed on the Illumina NextSeq system using 2 × 75bp paired-end sequencing. The RNA Seq data can be found in the NCBI GEO database under the accession number: GSE102364.

### 2.13. Bioinformatics

Sequencing reads were aligned to the Human GRCh38 genome using Hisat2 [37]. Expression value for FPKM and TPM for each gene based on Ensembl genome annotation was calculated using StringTie [38]. Differential expression of genes between samples was performed using Cuffdiff with statistical cut-off at |log-fold-change| > 1 and FDR < 0.1 [39]. J1.1 reads were aligned against the HIV-1 HBX2 reference genome using Geneious R11 software. Nucleotides that did not match the reference genome were highlighted.

### 2.14. Mitochondrial DNA PCR

PCR amplification has been performed from extracted DNA from EVs. Two sets of PCR-amplification primers designed for hyper variable (HV) regions of mtDNA HV2 and HV3 with respectively 374 and 232 base pairs amplicons were used [40,41]. Set-I: HV2F (5′- CACCCTATTAACCACTCACG-3′), HV2R (5’-CTGGTTAGGCTGGTGTTAGG-3′). Set-II: HV3F (5′-TCTTTTGGCGGTATGCACTTT-3′), HV3R (5’-GATGTGAGCCCGTCTAAACA-3′). PCR was performed using Q5 High-Fidelity PCR Kit (New England Biolabs) under the following conditions: 98 °C for 30 s; 35 cycles of 98 °C for 10 s, 62 °C for 30 s, and 72 °C for 30 s; and 72 °C for 2 min. The PCR product has been detected on 1.5% Agarose gel.

## 3. Results

### 3.1. Proteomic Analysis of Exosomes from HIV-1-Infected and Uninfected T-cells

In previous studies, we have observed that EVs from HIV-1-infected and uninfected cells have significant biological effects on recipient cells [16,17,27]. For example, we have shown that EVs from uninfected cells reversed HIV-1 latency, leading to HIV-1 transcription and the generation of mostly non-coding, and some coding RNA viral products in infected cells [27]. Furthermore, the EVs from HIV-1-infected cells were able to regulate the PKR/eIF2α and TLR3/NF-κB pathways in recipient cells as well as downregulate apoptosis through Bim [16,17]. Here, we examined the EVs from both uninfected and HIV-1-infected cells to determine their unique protein content, which may have contributed to the functional responses we observed previously.

EVs were isolated using our ExoMAX/density gradient separation method described previously [36]. Briefly, equal amounts of cell supernatant and ExoMAX reagent were mixed together and incubated overnight at 4 °C. EVs were precipitated out of solution following a 1500× *g* spin for 30 min the following day, resulting in an EV pellet. The pellet was then re-suspended in PBS and run on an iodixanol gradient. We then collected the 10.8 and 12.0 fractions, which have previously been shown to contain EVs (exosomes) positive for CD63, CD9, and CD81 [36], and used Nanotrap^®^ particles NT80/82 to capture the EVs away from the iodixanol. The EVs could then be trypsinized directly from the NT particles and mass spectrometry was performed in order to identify proteins. The amount of protein in each type of EV was evaluated semi-quantitatively based on the number of peptide sequences identified. For instance, if one EV had three-fold or more peptide sequences for one protein identified compared to other EVs, that protein was considered to be significantly upregulated.

Proteomic analysis was first conducted on EVs in the 10.8 fraction from uninfected CEM and HIV-1-infected ACH2 T-cells. A total of 1792 total proteins were identified through mass spectrometry, of which 384 were unique to uninfected EVs and 577 were unique to infected EVs (Figure S1). A number of proteins that were considered significant due to their presence as controls and/or regulation of viral spread are illustrated in Table 1. We found that both types of EVs contain HSP70 and HSP90, heat shock proteins (controls) that are well known markers of EVs and important carriers of proteins into EVs [42,43,44,45]. Furthermore, uninfected EVs contained 14-3-3 Protein while infected EVs contained nucleophosmin. Both types of EVs contained a number of heterogeneous nuclear ribonucleoproteins (hnRNPs), a type of RNA-binding protein important for sequence specific delivery of RNA into EVs [36,45,46]. However, infected EVs contained upregulated amounts of hnRNPs as compared to uninfected EVs. Likewise, infected EVs contained increased amounts of histones as compared to uninfected EVs. Interestingly, infected EVs contained all of the core histones, which suggests that these EVs may, in fact, contain a full nucleosome bound to DNA. In addition, infected EVs contained 15 cyclin-dependent kinases (Cdks), including Cdk9, which is important in transcription, and Cdks 1, 4, and 5, which are critical in cell cycle regulation [47,48,49,50]. There were, however, no detectable cyclin partners present in EVs. Additionally, infected EVs only contained 7 Src kinase family members, which are tyrosine kinases involved in a number of cellular processes, including cellular adhesion, cell motility, and T-cell signaling [51,52,53]. 

**Figure 1:**
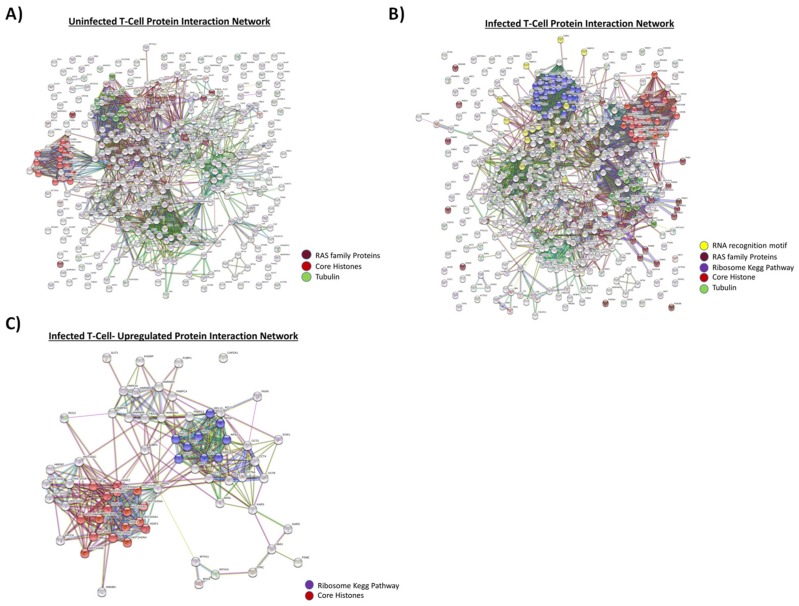
Protein comparison of uninfected and infected T-cell EVs. The 5-day old cell supernatants from CEM (uninfected) and ACH2 cells (HIV-1-infected) were harvested, treated with ExoMAX overnight, and centrifuged. The resulting pellet was run on an iodixanol density gradient, and the 10.8 fraction (exosome fraction) [41] was treated with NT80/82 overnight. The resulting pellet was treated was then prepared for mass spectrometry, and the resulting peptides were identified using Proteome Discoverer software. The predicted protein–protein interactions were then generated following multiple proteins input into the STRING database. A high confidence cutoff of ≥0.70–0.90 was implemented in this work. Network nodes represent proteins. Edges represent protein-protein associations and color shows association types. (**A**) Protein interaction network of proteins derived from uninfected T-cell exosomes (CEM). (**B**) Protein interaction network of proteins derived from infected T-cell exosomes (ACH2). (**C**) Protein interaction network of proteins which are upregulated in infected T-cell exosome.

These results are further illustrated using protein networks, which have become a popular tool for analyzing and visualizing the often-long lists of proteins or genes obtained from proteomics and other high-throughput technologies. One such network is the STRING database, which provides protein networks for more than 2000 organisms, including both physical interactions from experimental data and functional associations from curated pathways and prediction methods. The protein network of uninfected EVs consisted of 384 nodes and 1791 edges. These were mainly core histones, tubulin, and Ras family proteins (Figure 1A). In comparison to this, the protein network of the infected EVs was composed of 443 nodes and 3242 edges (Figure 1B). The infected protein interaction network mainly consisted of ribosome Kegg Pathway proteins, histones, RNA recognition motifs, tubulins, and Ras family proteins. When infected EVs were compared with uninfected EVs, there were 37 upregulated genes in infected T-cells which were identified as hub genes involved in RNA binding, ribosomal Kegg Pathway proteins, and core histones (e.g., RPL 12, GAPDH, HIST1H4H, etc.) (Figure 1C). Collectively, these results imply that there are clear protein cargo differences between EVs from uninfected and infected T-cells, possibly leading to different functional outcomes in the recipient cells. 

### 3.2. Functionality of EVs from HIV-1-Infected and Uninfected T-Cells

**Figure 2:**
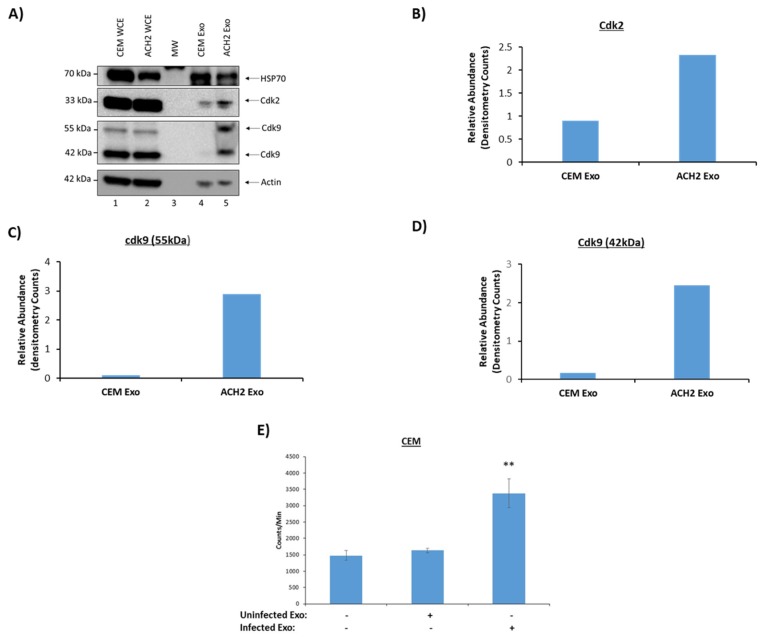
Infected T-cell have functional effects on recipient cells. The 5-day old cell supernatants from CEM (uninfected) and ACH2 cells (HIV-1-infected) were harvested, treated with ExoMAX overnight, and centrifuged. The resulting pellet was run on an iodixanol density gradient, and the 10.8 fraction (exosome fraction) [36] was treated with NT80/82 overnight. (**A**) Samples were Western blotted for HSP70 (control), Cdk2, Cdk9 (two isoforms 42 kDa and 55 kDa), and actin (control). Densitometry counts normalized to actin across all samples are shown for Cdk2 (**B**), Cdk9 55 kDa isoform (**C**), and Cdk9 42 kDa isoform (**D**). (**E**) Isolated EVs were added to recipient uninfected CEM cells, which had been synced at G0 phase, at a ratio of 1 cell:500 EVs and incubated for 44 h. [^3^H] thymidine was incorporated into the cells during a 4 h incubation. Cells were then washed and counted in a beta-counter to determine DNA synthesis. Student’s *t*-test compared untreated cells with cells treated with exosomes. ** *p* < 0.01, *Error bars*, S.D.

We next attempted to confirm some of the key results described in Table 1 using Western blots. Our results showed the presence of HSP70 in EVs from both cell types while Cdk2 and Cdk9 (two isoforms 55 kDa and 42 kDa [54,55,56]) were increased in infected EVs (Figure 2A). The increased expression of the Cdks were confirmed by quantification of the Western blot bands by densitometry analysis (Figure 2A–D). Specifically, there was a ~2.5-fold increase of Cdk2 expression between uninfected and infected exosomes, while both isoforms of Cdk9 (42 kDa and 55 kDa), increased by a ~28 and ~15-fold, respectively (Figure 2B–D). Although both were barely detectable from the uninfected EVs when using Western blots, mass spectrometry analysis did not show the presence of these proteins. This indicates that the Western blots may be slightly more sensitive than mass spectrometry analysis. 

Data in Table 1 and Figure 2A revealed that Cdk2, which is involved in cell cycle, and Cdk9, a protein involved in transcription, were both increased in EVs from infected cells [48,49,50]. This has implications in regard to cell cycle as Cdk2 is known to promote cell cycle progression [49,50]. Therefore, we performed a functional assay to assess whether the EV-related Cdks would indeed promote cell cycle progression in a recipient cell. Briefly, uninfected CEM cells blocked at G0 were incubated with either uninfected or infected EVs (1:500 ratio) and [^3^H] thymidine was incorporated into their DNA in order to quantify any new DNA synthesis. Results in Figure 2E indicate that the addition of uninfected EVs had minimal effect on DNA synthesis in the recipient cells; however, EVs from infected cells which had increased levels of Cdks, showed a significant increase in the amount of DNA synthesis observed in the recipient cells. Collectively, these data indicate that infected EVs from T-cells may promote cell cycle progression in the recipient cells.

### 3.3. Proteomic Analysis of Exosomes from HIV-1-Infected and Uninfected Monocytes

We also performed proteomic analysis on EVs from uninfected U937 and HIV-1-infected U1 cells. A total of 1311 proteins were identified through mass spectrometry, of which 552 were unique to uninfected monocytes and 274 were unique to infected monocytes (Figure S2). Proteins of interest are illustrated in Table 2. Similar to T-cell EVs, we found HSP70 and HSP90 in EVs from monocytes, confirming their presence as relevant EV markers. A number of proteins, including TGF-β Binding Protein 4 and ATP-citrate synthetase, were found to be unique to uninfected EVs while other proteins, such as Nucleolin and Interleukin Enhancer-BF3 were found in only infected EVs. Interestingly, monocyte EVs contained a number of complement-related proteins, including C3, C5, and C9. We also found a number of kinases in both uninfected and infected monocyte-derived EVs, including pyruvate kinase (involved in glycolysis) and NDK (involved in the exchange of terminal phosphate between NDPs and NTPs). However, there were a few kinases unique to uninfected and infected monocyte-derived EVs. For instance, uninfected EVs contained PI3K, a kinase involved in PI3K/AKT/mTOR signaling as well as autophagy [57], and cAMP kinase (PKA), which regulates several metabolic functions [58], while infected EVs contained PKR, an innate immune protein that binds double-stranded RNA [59]. Other comparisons showed a similarity to T-cell EVs in that EVs from infected monocytes contained an increased amount of hnRNPs. Interestingly, upon addition of cART, EVs from infected cells contained decreased amounts of hnRNPs. In fact, hnRNPs R, Q, K, U, and DL, which were found in EVs from infected cells but not uninfected cells, were absent in EVs from infected cells treated with cART [41]. Additionally, all core histones were found in EVs from infected cells, indicating that there may be nucleosomes contained in the EVs. However, infection did not seem to increase the number of histones in EVs when compared to uninfected cells. 

**Figure 3:**
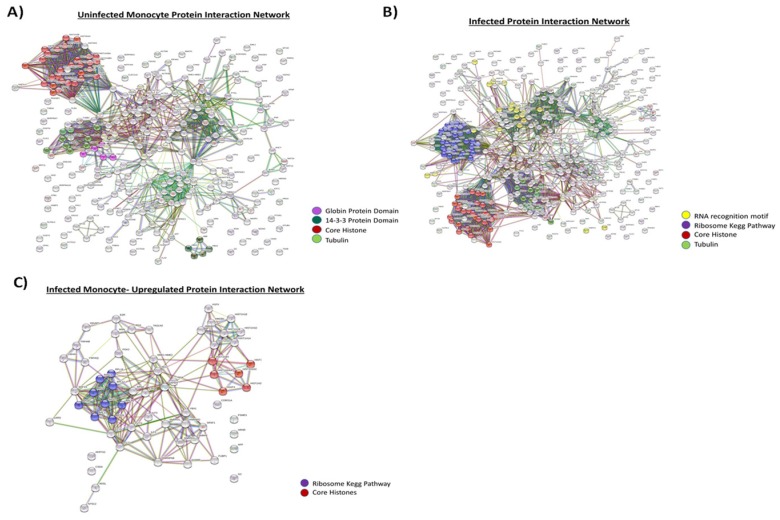
Protein comparison of uninfected and infected monocyte EVs. 5-day old cell supernatants from U937 (uninfected) and U1 cells (HIV-1-infected) were harvested, treated with ExoMAX overnight, and centrifuged. The resulting pellet was run on an iodixanol density gradient, and the 10.8 fraction (exosome fraction) [36] was treated with NT80/82 overnight. The resulting pellet was treated was then prepared for mass spectrometry, and the resulting peptides were identified using Proteome Discoverer software. The predicted protein–protein interactions generated following multiple proteins input into the STRING database. A high confidence cutoff of ≥0.70–0.90 was implemented in this work. Network nodes represent proteins. Edges represent protein-protein associations and color shows association types. (**A**) Protein interaction network of proteins derived from uninfected monocyte exosomes (U937). (**B**) Protein interaction network of proteins derived from infected monocyte exosomes (U1). (**C**) Protein interaction network of proteins which are upregulated in infected monocyte exosome.

These results are further illustrated using protein networks. An uninfected EV-derived protein network constructed by STRING consisted of 274 nodes and 1424 edges. These were mainly core histones, tubulin, and proteins with 14-3-3 and globin domain (Figure 3A). In contrast, the infected EV-derived protein network was composed of 379 nodes and 3114 edges (Figure 3B). The infected protein network mainly consisted of ribosome Kegg Pathway proteins, histones, RNA recognition motifs, and tubulins. When infected EVs were compared with uninfected EVs, there were 35 upregulated genes in infected monocytes, which were identified as hub genes involved in RNA binding, ribosomal Kegg Pathway Proteins, and core histones (e.g., RPL 12, GAPDH, HIST1H4H etc.) (Figure 3C). Collectively, these results imply that there are some differences between EVs from uninfected and infected monocytes, possibly leading to varying functional differences between the two types of EVs.

### 3.4. Functionality of EVs from HIV-1-Infected and Uninfected Monocytes

**Figure 4:**
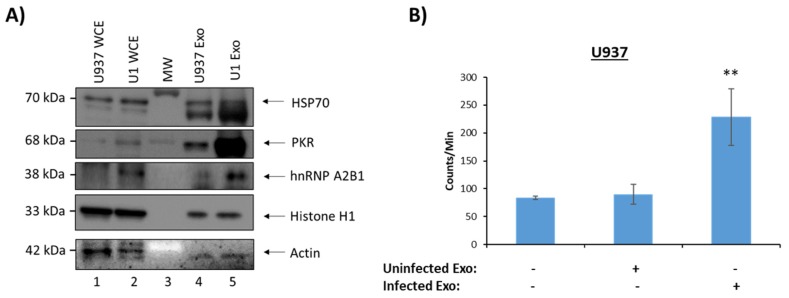
Infected monocyte EVs functional effects on recipient cells. 5-day old cell supernatants from U937 (uninfected) and U1 cells (HIV-1-infected) were harvested, treated with ExoMAX overnight, and centrifuged. The resulting pellet was run on an iodixanol density gradient, and the 10.8 fraction (exosome fraction) [36] was treated with NT80/82 overnight. (**A**) Samples were Western blotted for HSP70 (control), PKR, hnRNPA2/B1, histone H1, and actin (control). (**B**) Isolated EVs were added to recipient uninfected U937 cells, which had been synced at G0 phase, at a ratio of 1 cell:500 EVs and incubated for 44 h. [^3^H] thymidine was incorporated into the cells during a 4 h incubation. Cells were then washed and counted in a beta-counter to determine DNA synthesis. Student’s *t*-test compared untreated cells with cells treated with exosomes. ** *p* < 0.01, *Error bars*, S.D.

We next performed a similar set of experiments as Figure 2 using infected and uninfected monocyte-derived EVs. We performed Western blot analysis on several proteins of interest. Our results showed HSP70 in EVs from both cell types, in addition to Histone H1, while PKR and hnRNPA2/B1 (a protein we observed previously in our EVs [41]) was confirmed to be present in very high levels in EVs from infected, but not uninfected cells (Figure 4A). A functional assay was then performed to assess whether EV-related HMGB1 protein, a DNA binding protein, would promote cell cycle progression in a recipient cell. Briefly, uninfected U937 cells blocked at G0 were incubated with uninfected or infected EVs, and [^3^H] thymidine incorporation into the genomic DNA was measured. Results in Figure 4B indicate that addition of uninfected EVs had minimal effect on DNA synthesis in the recipient cells. However, EVs from infected cells increased DNA synthesis in recipient U937 cells by a significant rate. This suggests that EV-associated HMGB1 may potentially promote cell cycle progression.

Collectively, our data points toward HIV-1 infection contributing to an increase of hnRNP packaging into EVs across different cell types, which is consistent with our previous findings [36]. This result suggests that HIV-1 infection could contribute to an increase in packaging of hnRNPs and potentially associated non-coding RNA in EVs from infected cells compared to uninfected cells. Furthermore, our data point to the possibility of DNA being present in EVs from infected T-cells and monocytes as all four core histones were present in these fractions. This is consistent with all 4 histones forming histone/DNA monomer, dimer, or octomer complexes [60,61]. Additionally, the presence of kinases in EVs (i.e., Cdks in EVs from infected T-cells and PI3K and PKA in uninfected monocyte EVs) may contribute towards EVs playing a role in transcription and cell cycle progression in recipient cells.

### 3.5. Proteomic Analysis of EVs from PBMCs

We next examined the effect of infection and EV production from infected primary cells. Briefly, we cultured PBMCs from three different donors for five days prior to collecting the cell supernatant and isolating total EVs by using ExoMAX precipitation. The PBMCs were then infected with 89.6 dual-tropic HIV-1 (MOI: 10) and moved toward latency using cART and IL-7 treatment over the course of 7 days. Cell supernatants were then harvested from the infected cells and total EVs were isolated using ExoMAX precipitation. Mass spectrometry was then performed for both the EVs from uninfected and infected PBMCs. The amount of protein in each type of EV was evaluated semi-quantitatively based on the number of peptide sequences identified. If one EV had twofold or more peptide sequences for one protein identified compared to other EVs, that protein was considered to be significantly upregulated. Furthermore, semi-quantitative analysis across the three PBMCs was performed by averaging the number of peptide sequences recognized and comparing the resulting numbers across the two treatment groups.

**Figure 5:**
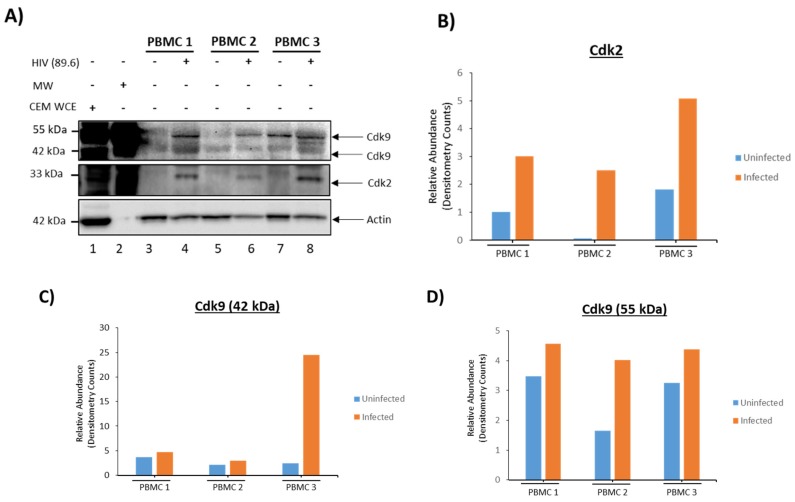
Infected PBMC EVs have increased levels of Cdk9. 6-day old cell supernatants from uninfected PBMCs was harvested and treated with ExoMAX overnight. PBMCs were then infected with HIV-1 89.6 (MOI 10) and placed under cART. 7-day old cell supernatants from the infected PBMCs were harvested and treated with ExoMAX overnight. Samples were spun down, re-suspended in PBS, and loaded on a gel. Western blot analysis for Cdk9 (two isoforms 42 kDa and 55 kDa) and actin (control) was performed on the PBMC EVs (**A**). Densitometry counts normalized to actin across all samples are shown for Cdk2 (**B**), Cdk9 42 kDa isoform (**C**) and 55 kDa isoform (**D**).

Two thousand-four hundred-fifty one proteins were identified through mass spectrometry from uninfected PBMC EVs. A number of proteins that we deemed significant are illustrated in Table 3. All EVs contained HSP90, confirming successful capture of EVs. Interestingly, HSP90 was found to be upregulated in EVs from all 3 uninfected PBMCs compared to EVs from infected PBMCs. Furthermore, talin-1 was identified in all EVs while integrin α-II preprotein was found only in EVs from uninfected cells and sodium/calcium exchanger 3 preprotein was found only in EVs from infected cells. Unlike in EVs from infected cell lines, we found that EVs from infected PBMCs generally contained less histones compared to the EVs from uninfected PBMCs. Additionally, EVs from uninfected PBMCs contained upregulated annexins when compared to EVs from infected PBMCs. However, EVs from infected PBMCs showed upregulation of hnRNP U, which is similar to the effect seen in Figure 1A and Figure 2A where hnRNPs were generally upregulated in EVs from infected cells. This suggests that EVs from infected PBMCs may contain more RNA than EVs from uninfected PBMCs. Furthermore, the lack of histones in EVs from infected PBMCs may be explained by these PBMCs being normal cells whereas the cell lines are cancer cells, and the dynamics of histone packaging into EVs may be different in infected normal cells compared to infected cancer cells. We also observed the presence of actin dynamic proteins, profilin and cofilin, in PBMC EVs, which may influence actin dynamics in recipient cells. In an HIV-1-infected environment, this could have implications in regard to viral spread [62]. Additional Western blot analysis for the presence of Cdks, which we previously implicated in functional effects of EVs in recipient cells (Figure 2), revealed Cdk2 and Cdk9 in EVs from both uninfected and infected PBMCs (Figure 5A). When densitometry was performed and results normalized to actin, we saw a dramatic increase of Cdk2 and Cdk9 (isoforms 42 kDa and 55 kDa) in EVs from infected cells (Figure 5B–D). This implies that infected EVs from PBMCs, like those from cell lines, could elicit functional effects such as increased transcription or potentially cell cycle progression in recipient cells [27]. Collectively, these data indicate that HIV-1-infection of primary cells significantly alters protein content of EVs where sodium/calcium exchanger 3 preprotein could potentially be used as a marker to examine a normal versus infected state.

### 3.6. EVs from HIV-1-Infected Cells Contain Similar Size RNAs

**Figure 6:**
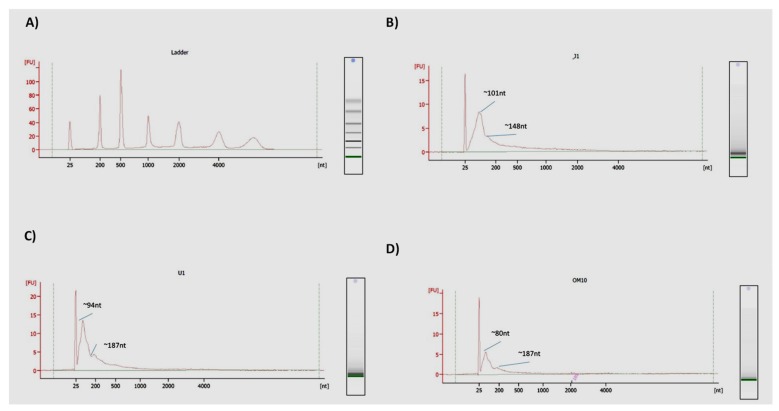
RNA Agilent Trace test shows RNAs of similar size in EVs. (**A**) RNA ladder was run as a control for an RNA Agilent Trace test. EVs from 5-day old J1.1 (infected T-cell) (**B**), U1 (infected monocyte) (**C**), and OM10.1 (infected myeloid) cells (**D**) were isolated by NT80/82 pulldown. Total RNA was isolated from the EVs and RNA Agilent Trace was performed to determine the peak size of RNA within the EVs.

To determine the size distribution of the RNAs from infected cell EVs, we utilized an RNA-seq approach. We have previously shown that HIV-1 TAR RNA, a double-stranded hairpin loop of 23 base pairs which binds to HIV-1 Tat protein, is found in EVs from infected cells [16,17,36]. Furthermore, we recently found that a novel less abundant long non-coding RNA, termed TAR-*gag*, is also found in EVs from infected cells [27]. We therefore asked whether there were other cellular or viral RNAs in EVs from infected cells. Briefly, EVs were isolated from 5-day-old infected cell supernatant and pulled-down using NT80/82. Total RNA was then isolated from the NTs and RNA sequencing was performed. Results from RNA Agilent Trace in Figure 6 show that EV-associated RNA from J1.1 (Figure 6B), U1 (Figure 6C), and OM10.1-derived EVs (Figure 6D) contain peak RNA lengths of ~101, 94, and 80 nucleotides, respectively. An additional peak RNA length occurred at 148 nucleotides for J1.1 EVs and 187 nucleotides for U1 and OM10.1 EVs. This indicates a potential as of yet unidentified machinery for specific RNA processing/and packaging into EVs, and a slight modification of packaging between myeloid and T-cells. Collectively, these data indicate that RNAs of about ~100 nucleotides, on average, are preferentially packaged into EVs, where they could contain small viral RNAs, miRNAs, and other short or long non-coding RNAs.

### 3.7. EVs from HIV-1 Infected T-Cells Contain Wild-Type and Modified Antisense RNA Transcripts

**Figure 7:**
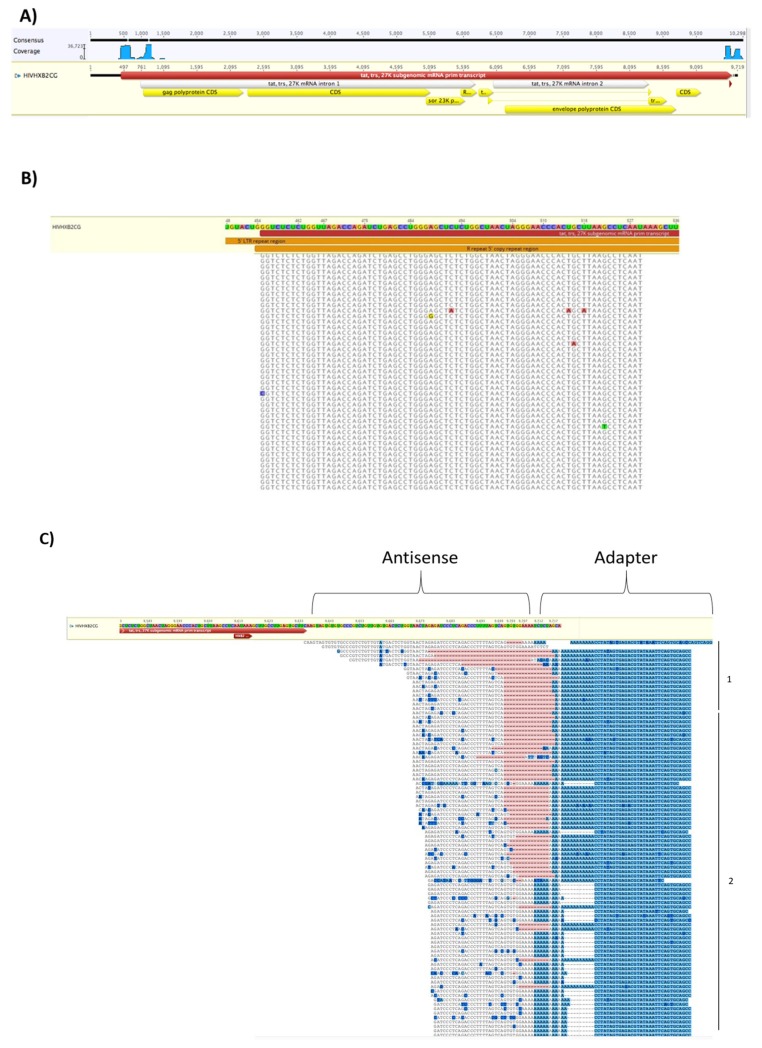

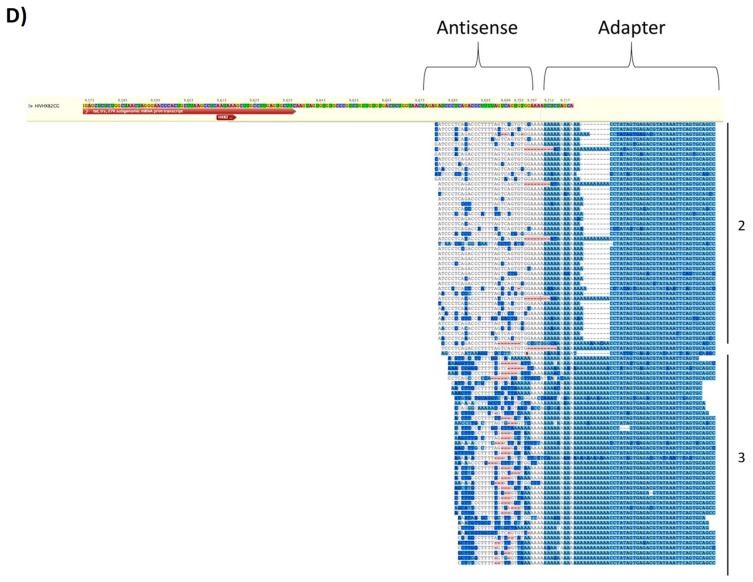
RNAseq bioinformatics analysis of EV-associated RNA. EVs from 5-day old J1.1 (infected T-cell) were isolated by NT80/82 pulldown and subjected to RNA sequencing. Bioinformatics analysis was performed using Geneius R11. Highlighted nucleotides represent deviations from the reference genome. (**A**) Bioinformatics analysis shows high amounts of HIV-1 RNAs at the 5′ of the HIV-1 genome and at the 3′ end of the HIV-1 genome. (**B**) Read-throughs of the 5′ end of the HIV-1 genome show sense RNAs that are very similar to the reference genome. (**C**) Read-throughs of the 3′ end of the HIV-1 genome show antisense RNAs that have some deviations from the reference genome. These RNAs fall into two possible groups. Longer RNAs (1) may be associated with wild-type AST RNA [63] while the shorter RNAs may be associated with a separate set of noncoding RNAs (2). (**D**) Read-throughs of the 3′ end of the HIV-1 genome show antisense RNAs that have many deviations from the reference genome. RNAs with less deviations may be associated with Transcript 2 while RNAs with more deviations may be associated with a different type of transcript altogether (3).

We then utilized bioinformatics on our RNA sequencing study, separating out HIV-1 RNAs from cellular RNAs by aligning RNAs to the Human GRCh38 genome using Hisat2 and excluding them from further analysis. Viral RNA was then analyzed using Geneious R11 software by aligning EV-associated RNA transcripts to the viral HIVHXB2CG reference genome (Figure 7A). Geneious identified a number of transcripts associated with the 5′ LTR at the R, U5, and *gag* regions of the HIV-1 genome as well as the U3 region of the genome. The transcripts occurring at the R, U5, and *gag* regions include TAR and TAR-*gag*, consistent with previous findings [27]. Figure 7B represents the TAR sequence identified at the 5′ end of each RNA. Highlighted nucleotides represent aberration from the reference genome. Geneious calculated that there was a 99.5% match between the sequence read-throughs and the reference genome, indicating very close sequence homology. However, other antisense transcripts associated with the U3 region of the HIV-1 genome were also identified ((1), which may be associated with HIV-AST [63]; (2) and (3), which may be associated with short antisense HIV-1 RNAs), as shown in Figure 7C,D. Many of the nucleotides in the 3′ transcripts are highlighted in comparison to the 5′ transcripts, indicating higher deviation from the reference genome. This suggests the short RNA molecules may be mutated at a higher rate. These mutations, however, might not be due to actual base change, but due to post-transcriptional modification of the RNA, such as methylation. Previous studies, for example, have shown that a number of modifications, in particular methylation, occur at the 3′ end of the HIV-1 genome [64,65]. Collectively, these results indicate that EVs from HIV-1-infected cells contain not only RNA from the 5′ LTR but also a mixture of wild type and mutant short RNAs from the 3′ LTR.

### 3.8. EVs from Infected Cells Contain Cellular Nucleic Acids

As indicated previously, EVs isolated from HIV-1-infected T-cells and myeloid cells contained cell-derived RNAs. RNA sequencing was performed and a number of cellular RNAs were identified using Ensembl. More than 10,000 RNAs were identified in T-cell EVs while less than 700 were identified in myeloid-derived EVs with TAR RNA functioning as a control. RNAs associated with MSI2, SON, ADAM10, and Gak were found in EVs from both cell types (Table 4a). Three thousand-sixty-five RNAs were unique to T-cell EVs, including several long non-coding RNAs (Table 4b). One hundred-thirty-six RNAs were unique to myeloid EVs, a number of which were interferon-related (Table 4c). Furthermore, we found a number of miRNAs in our EVs. Sixty-eight miRNAs were found to be unique to T-cell EVs while three were found to be unique to myeloid EVs. T-cell-related miRNAs were found to be involved in transcription regulation and cell cycle regulation (i.e., miR-324 and miR-31) [66,67] while miR-155HG, which can downregulate IRAK3 [68], was found in myeloid EVs (Table 5).

We previously observed a number of histones in EVs from infected cells (Table 1 and Table 2). Because all four core histones were found in these EVs, we hypothesized that there was DNA within the EVs. In order to test this hypothesis, we captured EVs by NT80/82 pulldown from uninfected (CEM, Jurkat, U937) and HIV-1-infected (J1.1 and U1) cells. We also isolated EVs from HUT102 and MT2 cells, both of which are T-cells infected with HTLV-1. The EVs were then lysed and treated with RNase to remove RNA contaminants prior to phenol/chloroform with isopropyl alcohol treatment to separate any DNA from the protein. High salt concentration and isopropanol were used to precipitate DNA, which was dried and re-suspended in nuclease-free water. The re-suspended solution was run on an agarose gel, and the resulting bands from the EV-resuspension were confirmed to be DNA when compared to the positive control (data not shown). We then performed PCR analysis on mitochondrial DNA as this type of DNA has been shown to be present in EVs [69,70]. This analysis showed the presence of mitochondrial DNA from both hypervariable region 2 and hypervariable region 3 in EVs from all tested cell types (Figure S3). However, we had not excluded larger EVs, such as microvesicles, from this experiment since the samples were not filtered through a 0.22 μm filter prior to NT80/82 pulldown. Therefore, we repeated the previous experiment using ExoMAX/iodixanol gradient separation, isolating DNA from only the 10.8 and 12.0 fractions, which we previously have shown to contain exosomes and are not contaminated with HIV-1 virions [36]. When we focused on only EVs recovered from these two fractions, we found no mitochondrial DNA from either of the hypervariable regions in these EVs (Figure S4). Therefore, we concluded that mitochondrial DNA was associated with larger, denser EVs (i.e., microvesicles) and not exosomes. Collectively, our data indicate that cellular nucleic acids, including miRNA and mitochondrial DNA, can be found in EVs from uninfected, HIV-1-infected, and/or HTLV-1-infected cells.

## 4. Discussion

Extracellular vesicles, including exosomes, can carry protein and RNAs between cells, which may contribute to signal transduction in the recipient cell [17,20,27,71]. Our previous studies show a role for EVs in pathogenesis of HIV-1 as exosomes from infected cells can spread viral RNAs and proteins to neighboring uninfected cells [16,17]. Additionally, we have demonstrated that exosomes from uninfected cells can cause activation of latent HIV-1 in infected cells, which causes HIV-1 viral products to appear in the EVs subsequently produced by these infected cells [27]. Similarly, Arenaccio, et al. showed that exosomes from infected cells can also elicit viral activation in latent HIV-1-infected cells [34]. Our data show that there are differences in the protein and RNA compositions of EVs from both uninfected and infected cells, and that these EVs can elicit different functional responses in recipient cells depending on whether they came from an infected or uninfected cell.

Proteomics data using mass spectrometry showed that EVs from HIV-1-infected T-cells, when compared to EVs from uninfected T-cells, are enriched in histones, RNA-binding proteins (specifically hnRNPs), Cdks, and Src family kinases (Figure 1). This was further confirmed by Western blot analysis (Figure 2A) and indicates that infected EVs, due to the increased amount of Cdks, can increase DNA synthesis in recipient cells, thus pushing the cells through the cell cycle (Figure 2E). This is similar to EVs from U1 cells (HIV-1-infected monocyte), which contain HMGB1, a protein mainly involved in DNA-binding and transcription but also implicated in cell cycle progression [72,73]. As with the infected T-cell EVs, infected monocyte EVs were found to be enriched in histones and RNA-binding proteins (specifically hnRNPs) compared to EVs from uninfected cells (Figure 3A and Figure 4A), and they also increase synthesis of DNA in recipient cells (Figure 4B). However, it could be possible that EV-associated proteins such as Cdks and HMGB1 are only partially responsible for the observed increase in DNA synthesis in recipient cells; alternatively, or additionally, the increase in DNA synthesis could be due to a process called endoreduplication, in which cells do not undergo mitosis and, instead, initiate another round of DNA replication, leading to cells with extra chromosomes [74,75,76]. Consequently, this is a phenomenon often observed in cancer cells, specifically linked to mutations in p53 and Rb [74]. Future experiments will determine whether endoreduplication is truly taking place or whether the observed increase in DNA synthesis upon EV addition to recipient cells is solely due to EV-associated proteins. We also examined the protein content of EVs from uninfected and infected EVs from PBMCs from three different donors (Table 3). In general, unlike in EVs from cell lines, EVs from infected PBMCs had less histones than EVs from uninfected PBMCs. This could be because the dynamics of histone packaging into EVs may be different in infected normal cells compared to infected cancer cells. Additionally, profilin and cofilin, proteins that are involved in actin dynamics, are enriched in infected PBMC EVs and uninfected PBMC EVs, respectively. This may allow EVs to influence actin dynamics in recipient cells, which could have implications in regard to viral spread. In fact, uninfected PBMC EVs, which are enriched in cofilin compared to EVs from infected cells, may allow for increased susceptibility to viral infection in uninfected recipient cells as cofilin has previously been implicated in HIV-1 entry into cells [62,77]. Infected PBMC EVs also contain increased levels of Cdk9, which may allow for increased transcription in recipient cells and/or cell cycle progression.

A second area of our study examined the nucleic acid content of EVs from HIV-1-infected cells. RNA analysis (using Agilent) revealed that EV-associated RNA had a peak size of around 100 nt in length (Figure 6). This indicates that there is a specific processing mechanism for RNA prior to its packaging into an EV, as evident by presence of hnRNPs in infected EVs, which play a significant role in the packaging process (Table 1 and Table 2), consistent with previous results [36]. RNA sequencing from EVs demonstrated several clusters of RNA, including short RNA at the beginning of the 5′ end of the HIV-1 genome and longer RNA of around 615 nt long. These RNAs include HIV Transactivating Response Element (TAR), a short double hairpin-looped RNA loop of about 23 bp, and a novel noncoding RNA we recently discovered and characterized called TAR-*gag* [17,27,78,79]. Interestingly, there was also a cluster of RNAs at the 3′ end of the HIV-1 genome; these RNAs were found to be antisense in nature and some showed a high level of mutation (Figure 7). These mutations could be due to poor proofreading of RNA polymerase II and polymerase pausing at the 3′ end [80,81]. As the HIV-1 genome contains a 3′ TAR molecule, it is possible that these antisense transcripts are double hairpin-loop structures [82]. Therefore, these antisense transcripts could have a functional effect in recipient cells that is similar to TAR, in that they can suppress innate immunity, activate TLR3 (or other TLRs), and induce NF-κB-mediated cytokine production [16,17]. Alternatively, these molecules could be modified by RNA editing, such as A-to-I modifications, which are driven by adenosine deaminase [83], a protein we found in EVs from infected monocytes. Other potential possibilities include methylation modifications, such as m6A, which could allow for misincorporation of bases at the time of cDNA synthesis during library preparation. In this case, these RNAs may function similarly to TAR-*gag* in that they act as a RNA scaffold to bind proteins that lead to different functional effects [79]. Future studies will better define the function of the 3′ antisense RNA; interestingly, recent data have shown that a large antisense transcript is able to regulate gene expression at the 5′ LTR [63].

Our RNA sequencing analysis also identified a number of cellular RNA transcripts in EVs from infected cells, including MSI2 and SON, which both code for RNA-binding proteins (Table 4). Interestingly, there were several RNAs unique to T-cell-derived EVs, including several long noncoding RNAs. We also found a number of interferon-related RNAs which were unique to only monocyte EVs. Furthermore, we identified 68 miRNAs in T-cell EVs while only 3 miRNAs were identified in monocyte EVs (Table 5). In T-cell EVs, some of these miRNAs, such as miR-24 and miR-31, targeted apoptotic proteins, which may enable HIV-1 to promote a pro-cancer environment [67,84]. This could be further exasperated by presence of TAR RNA carried within these vesicles as TAR has recently been shown to directly promote cell growth and progression of cancer [18,27]. Monocyte EVs, on the other hand, carry miR-155, which has been implicated in the suppression of type I interferon [68]. This may suppress overall immune response, enhancing HIV-1′s pathogenesis as well as susceptibility to other types of infections. Additionally, we observed mitochondrial DNA in EVs from uninfected T-cells and monocytes, HIV-1-infected T-cells and monocytes, and HTLV-1-infected T-cells (Figure S3). This EV-associated DNA was affiliated with two mitochondrial hypervariable regions, HVR-2 and HVR-3. Interestingly, this DNA was not present in EVs from the 10.8 fraction only (Figure S4). This indicates that mitochondrial DNA is in denser, slightly larger EVs as opposed to less dense, slightly smaller EVs in the fraction 10.8. Collectively, our data indicates that EVs from uninfected and infected cells carry nucleic acids that may be involved in viral pathogenesis.

Overall, we have shown that infection of cells with HIV-1 leads to differences in cargo and functional effect of EVs. In cell lines, HIV-1 infection leads to increased histone and RNA-binding proteins present in EVs. Furthermore, EVs from infected cells allowed for enhanced cell cycle progression in recipient cells, which was likely due to the increased Cdks and HMGB1 proteins in T-cell and monocytes EVs, respectively. PBMC EVs, on the other hand, showed a different trend with histones being upregulated in EVs from uninfected cells whereas actin-related proteins, such as profilin, were upregulated in EVs from infected cells. This difference could be explained by differences in packaging mechanisms of EVs between cancer cells and normal cells, such as activated PBMCs. We also found that EVs from infected cells contained RNA with a peak length of about 100 nt and an antisense HIV-1 transcript that was heavily mutated at the 3′ end of the genome. These EVs also carried a number of cellular RNAs, including lncRNAs, miRNAs, some of which could be associated with dampening the host’s immune response and enhancing HIV-1 pathogenesis, and mitochondrial DNA. Taken together, we propose that EV-associated Cdks and EV-associated lncRNAs and miRNAs may work in tandem. In this model, EVs enter quiescent recipient, neighboring cells (G0 phase of the cell cycle) and EV-associated Cdks could then promote cell cycle progression, leading the recipient cell to enter G1, S, or G2 phase of the cell cycle. This would cause the cell’s DNA, which, in G0, is normally in a tight, transcriptionally-suppressed state, to become loose, promoting transcription of various genes. Then, EV-associated lncRNAs could further regulate transcription of genes important for cellular growth. Gene expression of cycling cells may loosen chromatin and allow non-coding RNA to better regulate promoter target sites. 

Our results are consistent with the notion that EVs from infected cells may contribute to the pathogenesis of HIV-1. These EVs contain proteins that can activate cell cycle, and in recipient uninfected T-cells and/or monocytes, this could allow for increased HIV-1 susceptibility [17,85]. Furthermore, these EVs have been shown to contain HIV-1 TAR, which causes immune dysfunction in uninfected recipient cells by inhibiting innate immune response via the PKR pathway and by inducing cytokine production via TLR-3 activation [17]. This increased cytokine production could lead to increased inflammation, which may lead to cell death [85]. This would be especially problematic in specific tissues, such as the CNS, where cell death mediated by EV-enhanced inflammation could contribute to HIV-1 Associated Neurocognitive Disorders. Additionally, our data could be significant to other diseases. Along these lines, a recent publication by Singh Chahar, et al. indicates functional differences between EVs from uninfected and infected cells in RSV infection, further highlighting the importance of this topic [86]. Furthermore, we found that EVs from HIV-1-infected cells contain a short anti-sense RNA transcript that are mutated. Future experiments will address whether it plays a role in pathogenesis (such as binding PKR and inhibiting its ability in recipient cells) [17] or functions as an RNA scaffolding molecule (an RNA machine). Overall, our data point to an alteration in EV cargo and functionality in uninfected recipient cells that could determine the fate of viral propagation.

## Figures and Tables

**Table 1:** Protein comparison of uninfected and infected T-cell extracellular vesicles (EVs).

	Protein	Uninfected T-cell Exosome (CEM)	Infected T-cell Exosome (ACH2)
**Controls**	Nucleophosmin	-	+++
	14-3-3 Protein	+	-
	HSP70	+	+
	HSP90	+	+
**RNA Binding Proteins**	hnRNP A1	-	+
	hnRNP L	-	++
	hnRNP R	+	++
	hnRNP D0	-	++
	hnRNP Q	+	+
	hnRNP K	+	-
**Histones**	Histone H1	+	++
	Histone H2A	-	++
	Histone H2B	+	+
	Histone H3	+	++
	Histone H4	-	+++
**Kinases (Cdks)**	Cdk1	-	+
	Cdk2	-	+
	Cdk3	-	+
	Cdk4	-	+
	Cdk5	-	+
	Cdk6	-	+
	Cdk9	-	+
	Cdk12	-	+
	Cdk13	-	+
	Cdk14	-	+
	Cdk15	-	+
	Cdk16	-	+
	Cdk17	-	+
	Cdk18	-	+
	Cdk19	-	+
	Cdk20	-	+
**Kinases (RTKs)**	Blk	-	+
	Fgr	-	+
	Fyn	-	+
	Hck	-	+
	Lck	-	+
	Lyn	-	+
	Yes	-	+

The 5-day old cell supernatants from CEM (uninfected) and ACH2 cells (HIV-1-infected) were harvested, treated with ExoMAX overnight, and centrifuged. The resulting pellet was run on an iodixanol density gradient, and the 10.8 fraction (exosome fraction) [36] was treated with NT80/82 overnight. The resulting pellet was treated was then prepared for mass spectrometry, and the resulting peptides were identified using Proteome Discoverer software. A table showing key protein differences between CEM and ACH2 EVs. HSPs are used as a control while differences between EVs are seen in RNA binding proteins, histones, Cdk kinases, and Src family kinases. The amount of protein in each type of EV was evaluated semi-quantitatively based on the number of peptide sequences identified. Proteins are denoted as being absent (“-“) or present (“+”). When the difference in the number of peptide sequences identified for a protein in each type of EV is less than 3×, both proteins are denoted as present (“+”). If a protein is increased between 3× and 6× in one EV type over the other, then the protein is denoted as enriched (“++”). If a protein is increased by at least 6× in one EV type over the other, then the protein is denoted as significantly enriched (“+++”).

**Table 2:** Protein comparison of uninfected and infected monocyte EVs.

	Protein	Uninfected Monocyte Exosome (U937)	Infected Monocyte Exosome (U1)
**Controls**	TGF-β binding protein 4	+++	-
	Nucleolin	-	+++
	HSP70	+	+
	HSP90	+	+
	Complement Protein C1	+	+
	Complement Protein C3	+	+
	Complement Protein C4A	+	+
	Complement Protein C5	+	+
	Complement Protein C7	+	-
	Complement Protein C8	+	+
	Complement Protein C9	+	+
	Complement Protein H	+	-
**RNA Binding Proteins**	hnRNP A1	-	++
	hnRNP R	-	+
	hnRNP D0	-	++
	hnRNP Q	-	+
	hnRNP K	-	+
	hnRNP U	-	+
	hnRNP DL	-	+
	hnRNP C1/2	-	+
	hnRNP A1/B1	-	+
	hnRNP A/B	-	+
**Histones**	Histone H1	-	+
	Histone H2A	-	+
	Histone H2B	+	+
	Histone H3	+	+
	Histone H4	+	+
**Kinases**	PKM	+++	+++
	PGK1	+	+
	NDK	+	+
	Int Kinase	+	+
	Adeno Kinase	+	-
	cAMP Kinase	+	-
	PI3K	+	-
	Xylulose Kinase	+	-
	BADDA	+	-
**Infected Cell Exosomes**	PKR	-	+
	HMGB1	-	++
	Interleukin enhancer-BF3	-	++
	FVEB1	-	++
	T-Complex Protein	-	++
	Adenylosuccinate lyase	-	++
	Guanine nuc-binding protein	-	++
**Uninfected Cell Exosomes**	Spondin-1	++	-
	ILGBP	++	-
	EMILIN-2	++	-
	ATP-citrate synthase	++	-
	Proteoglycan 4	+	-
	Exostosin-1	+	-

The 5-day old cell supernatants from U937 (uninfected) and U1 cells (HIV-1-infected) were harvested, treated with ExoMAX overnight, and centrifuged. The resulting pellet was run on an iodixanol density gradient, and the 10.8 fraction (exosome fraction) [36] was treated with NT80/82 overnight. The resulting pellet was treated was then prepared for mass spectrometry, and the resulting peptides were identified using Proteome Discoverer software. A table showing key protein differences between U937 and U1 EVs. HSPs are used as a control while differences between EVs are seen in RNA binding proteins, histones, kinases, and other proteins. The amount of protein in each type of EV was evaluated semi-quantitatively based on the number of peptide sequences identified. Proteins are denoted as being absent (“-“) or present (“+”). When the difference in the number of peptide sequences identified for a protein in each type of EV is less than 3×, both proteins are denoted as present (“+”). If a protein is increased between 3× and 6× in one EV type over the other, then the protein is denoted as enriched (“++”). If a protein is increased by at least 6× in one EV type over the other, then the protein is denoted as significantly enriched (“+++”).

**Table 3:** Protein comparison of uninfected and infected peripheral blood mononuclear cells (PBMC) EVs.

	Protein	Uninfected PBMC 1	Infected PBMC 1	Uninfected PBMC 2	Infected PBMC 2	Uninfected PBMC 3	Infected PBMC 3
**Controls**	Integrin Alpha-II B Preprotein	+++	-	+++	-	+++	-
	Sodium/Calcium Exchanger 3 Preprotein	-	++	-	++	-	++
	Talin-1	+	+	+	+	+	+
	HSP 90	++	+	++	+	++	+
**RNA Binding Proteins**	hnRNP K	+	+	++	+	++	+
	hnRNP C1/C2	+	+	+	+	+++	-
	hnRNP R	+	-	++	-	+++	-
	hnRNP Q	+	+	+++	+	++	+
	hnRNP U	+	+	+	+++	+	+++
	hnRNP D0	++	+	+++	+	+++	+
**Histones**	Histone H1	+++	-	+++	+	+++	+
	Histone H2A	++	+	++	+	++	+
	Histone H2B	+	+	++	+	+	+
	Histone H3	++	+	+++	+	+++	+
	Histone H4	+	+	+	+	+++	+
**Annexins**	Annexin A1	+++	+	++	+	+++	+
	Annexin A2	++	+	+++	+	++	-
	Annexin A5	+++	-	-	-	+	-
	Annexin A6	++	+	+++	+	+++	+
**Misc. Proteins**	Proflin	+	+	+	+++	+	+++
	Cofilin	+	+	++	+	+++	+
	Elongation Factor 1	++	-	+	+	++	-
	Elongation Factor 2	+	+	+	+	++	+
	Calponin 2	++	-	++	-	++	-
	Cathepsin G	+	-	+	-	-	-
	Plectin	+	-	+	-	+	-
	14-3-3 Protein Zeta/Delta	+	++	+	+	+	+
	Rap-1B	+	++	+	+	+	++
	HSP60	+	+++	+	+	+	+++

The 6-day old cell supernatants from uninfected PBMCs was harvested and treated with ExoMAX overnight. PBMCs were then infected with HIV-1 89.6 (MOI 10) and placed under cART. 7-day old cell supernatants from the infected PBMCs were harvested and treated with ExoMAX overnight. Mass spectrometry was performed on the EVs. The resulting peptides were identified using Proteome Discoverer software. The table shows differences between the 3 PBMC EVs pre- and post-infection. HSP 90 was used as a control while differences can be seen in RNA binding proteins, histones, annexins, and other proteins. The amount of protein in each type of EV was evaluated semi-quantitatively based on the number of peptide sequences identified. Proteins are denoted as being absent (“-“) or present (“+”). If a protein is increased by 2× in one EV type over the other, then the protein is denoted as enriched (“++”). If a protein is increased by at least 3× in one EV type over the other, then the protein is denoted as significantly enriched (“+++”). Proteins are absent, present, increased 2× over the other, or increased 3× over the other (“-“, “+”, “++”, “+++”), respectively.

**Table 4:** RNA comparison between T-cell and myeloid EVs.

(**a**)			
**RNA**	**Infected T-Cell Exosome**	**Infected Myeloid Exosome**	***P*-Value**
MS12	+	+	0.0097
SON	+	+	0.0054
ADAM10	+	+	0.0006
GAK	+	+	0.0107
(**b**)		(**c**)	
**Unique to T-Cell Exosome (3065 total RNAs)**		**Unique to Myeloid Exosome (136 total RNAs)**	
LINC00184		IFI44L (Interferon-Stimulated)	
LINC00152		IFI16 (Interferon-Stimulated)	
LINC01234		IRF8 (Interferon-Stimulated)	
LINC00969		SLFN11 (Interferon-Stimulated)	

EVs from 5-day old J1.1 and OM10.1 cell supernatant were isolated by NT80/82 pulldown. Total RNA was isolated from the EV and RNA sequencing was performed. (**a**) Table showing RNAs common to both T-cell and myeloid EVs. (**b**) Table showing RNAs unique to T-cell EVs. (**c**) Table showing unique RNAs to myeloid EVs.

**Table 5:** miRNA comparison between T-cell and myeloid EVs.

miRNA	Source	Target
miR-635	T-cell	ICAM-1
miR-324	T-cell	SP-1
miR-621	T-cell	FBXO11
miR-31	T-cell	p53
miR-590	T-cell	TGF-βR2
miR-221	T-cell	c-Kit
miR-25	T-cell	SRC-3
miR-29A	T-cell	CDK6
miR-24A	T-cell	INK4A
miR-34A	T-cell	SIRT-1
miR-155HG	Myeloid	IRAK3

EVs from 5-day old J1.1 and OM10.1 cell supernatant were isolated by NT80/82 pulldown. Total RNA was isolated from the EV and RNA sequencing was performed. The table shows miRNAs identified as being unique to T-cell EVs or myeloid EVs.

## Supplementary Materials

The following are available online at https://www.mdpi.com/2073-4409/8/8/787/s1, Figure S1: Number of proteins in EVs from uninfected and infected T-cells; Figure S2: Number of proteins in EVs from uninfected and infected monocytes; Figure S3: Mitochondrial DNA from hypervariable regions 2 and 3 is in EVs from infected and uninfected cells; Figure S4: Mitochondrial DNA from hypervariable regions 2 and 3 is not in EVs recovered from the 10.8 fraction.
