# Supplementary material for: An Omics Approach to Extracellular Vesicles from HIV-1 Infected Cells
Source: Cells. 2019 Jul 29;8(8):787. doi: 10.3390/cells8080787 (PMC6724219; doi:10.3390/cells8080787)
Supplement: Supplementary file 1 [file cells-08-00787-s001.pdf]

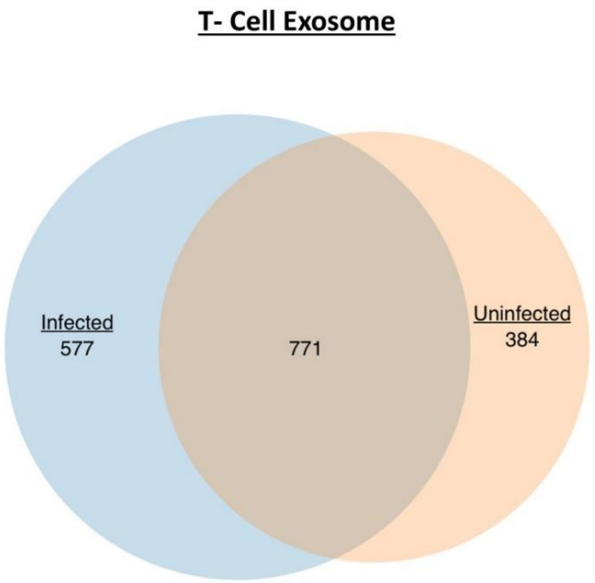

Figure S1: Number of proteins in EVs from uninfected and infected T-cells. 5-day old cell supernatants from CEM (uninfected) and ACH2 cells (HIV-1-infected) were harvested, treated with ExoMAX overnight, and centrifuged. The resulting pellet was run on an iodixanol density gradient, and the 10.8 fraction (exosome fraction) [41] was treated with NT80/82 overnight. The resulting pellet was treated was then prepared for mass spectrometry, and the resulting peptides were identified using Proteome Discoverer software. Using R, it was determined that 771 proteins were found to be in common between CEM and ACH2 EVs while 384 proteins were unique to CEM EVs and 577 proteins were unique to ACH2 EVs.

Monocyte Exosome

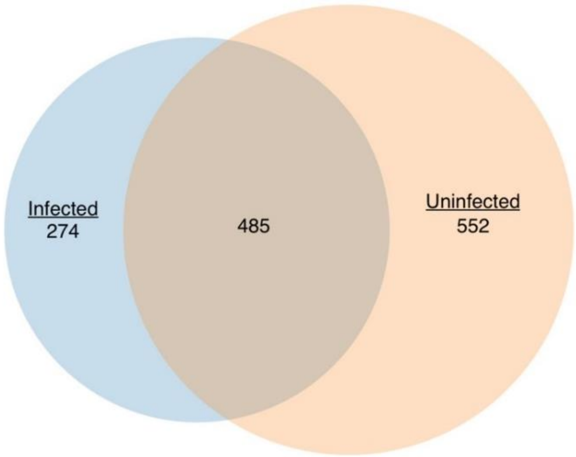

Figure S2: Number of proteins in EVs from uninfected and infected monocytes. 5-day old cell supernatants from U937 (uninfected) and U1 cells (HIV-1-infected) were harvested, treated with ExoMAX overnight, and centrifuged. The resulting pellet was run on an iodixanol density gradient, and the 10.8 fraction (exosome fraction) [41] was treated with NT80/82 overnight. The resulting pellet was treated was then prepared for mass spectrometry, and the resulting peptides were identified using Proteome Discoverer software. Using R, it was determined that 485 proteins were found to be in common between U937 and U1 EVs while 552 proteins were unique to U937 EVs and 274 proteins were unique to U1 EVs.

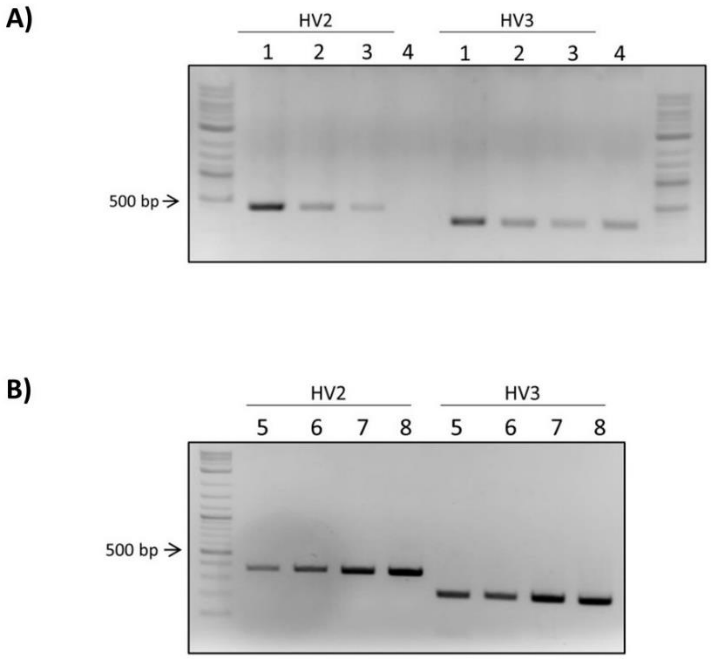

Figure S3: Mitochondrial DNA from hypervariable regions 2 and 3 is in EVs from infected and uninfected cells. 5-day old cell supernatants from ACH2 (1), U1 (2), HUT102 (3), J1.1 (4), MT-2 (5), Jurkat (6), CEM (7), and U937 (8) cells were harvested and treated with NT80/82 to isolate EVs. DNA was isolated from the EVs for PCR analysis prior to being run on a 1.5% agarose gel. ACH2, U1, HUT102, and J1.1 EVs were run on one gel (**A**) MT-2, Jurkat, CEM, and U937 EVs were run on a second gel (**B**).

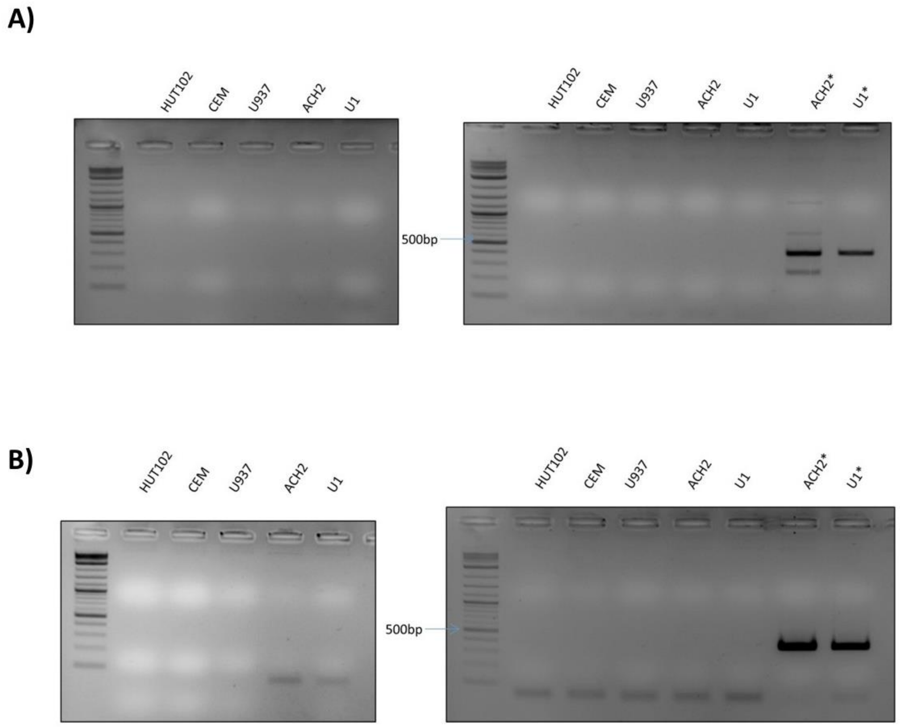

Figure S4: Mitochondrial DNA from hypervariable regions 2 and 3 is not in EVs recovered from the 10.8 exosome fraction. 5-day old cell supernatants from CEM, ACH2, HUT102, U937, and U1 cells were harvested, treated with ExoMAX overnight, and centrifuged. The resulting pellets were run on iodixanol density gradients, and the 10.8 fraction (exosome fraction) [41] was treated with NT80/82 overnight. DNA was isolated from the EVs and subjected to PCR analysis for mitochondrial DNA from hypervariable regions 2 **(A)** and 3 **(B)**. Samples marked by an asterisk represent DNA isolated from total EVs.
